# Neutrophil extracellular trap formation and circulating nucleosomes in patients with chronic myeloproliferative neoplasms

**DOI:** 10.1038/srep38738

**Published:** 2016-12-13

**Authors:** Cecilia P. Marin Oyarzún, Agostina Carestia, Paola R. Lev, Ana C. Glembotsky, Miguel A. Castro Ríos, Beatriz Moiraghi, Felisa C. Molinas, Rosana F. Marta, Mirta Schattner, Paula G. Heller

**Affiliations:** 1 Department of Hematology Research, Institute of Medical Research “Alfredo Lanari”, University of Buenos Aires, National Council for Scientific and Technological Research (CONICET), Buenos Aires, Argentina; 2 Laboratory of Experimental Thrombosis, Institute of Experimental Medicine (IMEX)- CONICET, National Academy of Medicine, Buenos Aires, Argentina; 3 Consultorios Hematológicos, Buenos Aires, Argentina; 4 Department of Hematology, Hospital Ramos Mejía, Buenos Aires, Argentina

## Abstract

The mechanisms underlying increased thrombotic risk in chronic myeloproliferative neoplasms (MPN) are incompletely understood. We assessed whether neutrophil extracellular traps (NETs), which promote thrombosis, contribute to the procoagulant state in essential thrombocythemia, polycythemia vera and myelofibrosis (MF) patients. Although MPN neutrophils showed increased basal reactive oxygen species (ROS), enhanced NETosis by unstimulated neutrophils was an infrequent finding, whereas PMA-triggered NETosis was impaired, particularly in MF, due to decreased PMA-triggered ROS production. Elevated circulating nucleosomes were a prominent finding and were higher in patients with advanced disease, which may have potential prognostic implication. Histone-MPO complexes, proposed as specific NET biomarker, were seldomly detected, suggesting NETs may not be the main source of nucleosomes in most patients, whereas their correlation with high LDH points to increased cell turn-over as a plausible origin. Lack of association of nucleosomes or NETs with thrombosis or activation markers does not support their use as predictors of thrombosis although prospective studies in a larger cohort may help define their potential contribution to MPN thrombosis. These results do not provide evidence for relevant *in vivo* NETosis in MPN patients under steady state conditions, although availability of standardized NET biomarkers may contribute to further research in this field.

Classic BCR-ABL-negative chronic myeloproliferative neoplasms (MPN) are stem cell disorders characterized by abnormal myeloid proliferation and increased blood cell counts and comprise polycythemia vera (PV), essential thrombocythemia (ET) and primary myelofibrosis (PMF)^1^. Although PV, ET and PMF share several biological and molecular features, including the fundamental role of JAK/STAT signaling in disease pathogenesis, their clinical presentation and natural course show distinctive features. Whereas PV and ET tend to be indolent conditions with a relatively long life expectancy, survival in PMF is significantly worse and patients may develop progressive extramedullary hematopoiesis and increased rate of leukemic transformation^1^.

Thromboembolic complications represent a major cause of morbidity and mortality in MPN, particularly in PV and ET, but also, albeit less frequently recognized, in PMF^2^,^3^. Age >60 years, previous thrombosis and the *JAK2*V617F mutation are established risk factors for thrombosis and prove useful for risk stratification^2^. However, accurate prediction of the thrombotic risk still represents a challenge and adequate predictive biomarkers are not available. The mechanisms underlying thrombosis are incompletely understood and involve a complex interplay among blood cells, the endothelium and the coagulation system^2^. Both increased blood counts and cellular activation, which is due to hyperactivation of intracellular signaling pathways and enhanced interaction between blood cells, are implicated in the thrombotic predisposition. The key role of leukocytes in the pathogenesis of MPN thrombosis has been highlightened by several studies^4^,^5^,^6^,^7^. Ongoing neutrophil activation, evidenced by increased membrane CD11b, release of proteolytic enzymes and neutrophil/platelet aggregates, contributes to the procoagulant phenotype^5^,^6^. Although several markers of leukocyte and platelet activation have been linked to thrombosis development, their value as predictors of thrombosis has not been established. In addition, a systemic proinflammatory state coexists with the MPN clone^8^. A wide array of proinflammatory cytokines released by malignant and non-malignant cells are elevated in the bloodstream and may exacerbate the prothrombotic state by activating platelets, endothelial cells and leukocytes^9^.

In recent years, neutrophils have been shown to release neutrophil extracellular traps (NETs), which are weblike structures composed of DNA and histones associated with granular proteins that contribute to innate immunity by entrapping microorganisms^10^. During NETosis, neutrophils decondense their chromatin, the granular and nuclear contents are mixed and NETs are released to the extracellular space following desintegration of the cellular membrane^11^. Reactive oxygen species (ROS) generated by the NADPH oxidase complex mediate most forms of NETosis^11^. Besides microorganisms, NETs may be triggered by a variety of stimuli, including cytokines, such as tumor necrosis alpha (TNFα) and interleukin (IL)-8^10^,^11^,^12^, activated platelets^13^,^14^ and autoantibodies^15^. Although primarily involved in host defense, NETs have been shown to promote thrombosis, as they provide suitable scaffolds for clot formation, binding red cells, platelets and von Willebrand factor (VWF), and induce a strong procoagulant response, mainly by contact phase activation and by enhancing tissue factor activity through elastase-mediated cleavage of tissue factor pathway inhibitor^16^,^17^. This process, which links innate immunity and thrombosis, has been named immunothrombosis^18^. The role of NETs in venous and arterial thrombosis has been demonstrated in animal models of experimental thrombosis^19^,^20^ and confirmed in human venous thromboembolism and coronary artery disease^21^,^22^, offering potential new targets for antithrombotic therapy. In this setting, circulating nucleosomes or cell-free DNA, presumed to originate from NETs, have been proposed as markers of thrombotic risk^23^.

Aside from their implication in thrombosis, excessive NET formation may trigger tissue damage and contribute to the pathogenesis of several disease states, as shown in sepsis^24^ and prothrombotic conditions characterized by sterile inflammation, such as systemic inflammatory response syndrome^12^, transfusion-related lung injury^13^, vasculitis^15^ and diabetes^25^. In addition, a role for NETs in neoplastic diseases has begun to be unraveled. Neutrophils from murine models of chronic myeloid leukemia, lung and breast cancer are sensitized towards NET generation^26^, suggesting NETs could be implicated in cancer-associated thrombosis.

Based on the presence of *in vivo* neutrophil activation in the setting of a chronic proinflammatory environment, we investigated whether NETs play a role in the thrombotic predisposition of MPN by studying the ability of MPN neutrophils to form NETs *ex vivo* and by measuring surrogate *in vivo* markers of NETosis.

## Results

### Patients

Patient features are detailed in Table 1 and reflect the variability in clinical phenotype found in daily clinical practice in patients with unselected PV, ET and myelofibrosis (MF), including primary, post-PV and post-ET MF. Plasma samples were available in 66 patients, including untreated patients and patients under different cytoreductive treatments, as detailed in Table 1. Assays involving neutrophils were performed in 32 of 66 patients, including untreated patients and patients treated with hydroxyurea. The number of patients included in each experiment is detailed where appropriate.

### NET formation by MPN neutrophils in resting conditions

**Figure 1:**
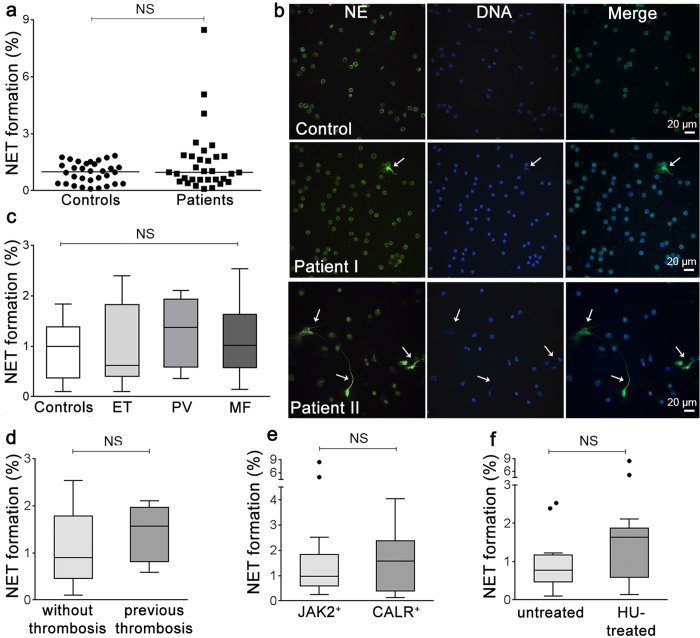
NET formation by unstimulated MPN neutrophils. (**a**) The percentage of neutrophils releasing NETs in the absence of NET inducers was scored by fluorescence microscopy in MPN patients (n = 32) and controls (n = 32). Horizontal lines represent median values. *P* = NS, Wilcoxon matched pairs test. (**b**) Representative images of NETs after staining with neutrophil elastase (NE)-specific antibody (green) and DNA stained with Hoechst dye (blue) in a healthy subject, a patient with normal NETosis (Patient I) and a patient showing increased spontaneous NET formation (arrows) (Patient II). Original magnification 40x. (**c**) Percentage of NETs according to MPN phenotype, including ET (n = 11), PV (n = 10) and MF (n = 11). Lines in the middle of the box are plotted at the medians, boxes are defined by the 25^th^ and 75^th^ percentiles and whiskers are drawn by the Tukey method. *P* = NS, Kruskal-Wallis test. (**d**) Unstimulated NET formation according to the absence (n = 23) or presence (n = 9) of previous thrombosis. *P* = NS, Mann-Whitney test. (**e**) Analysis of *JAK2*V617F^+^ (n = 21) vs. *CALR*^+^ (n = 7) patients. *P* = NS, Mann-Whitney test. (**f**) Comparison of untreated (n = 16) vs. hydroxyurea (HU)-treated (n = 16) patients. *P* = NS, Mann-Whitney test.

Based on the presence of neutrophil activation and increased thrombotic tendency, we determined whether MPN neutrophils were prone to release NETs spontaneously *ex vivo* by evaluating by microscopy NET production in the absence of NET inducers. NET formation by unstimulated neutrophils did not differ significantly between patients and controls (Fig. 1a). However, basal NET generation was heterogeneous among patients and increased NET release (higher than mean plus 2 SD of control values) was found in 6/32 (18.75%) patients, 2 with ET, 2 with PV and 2 with MF. Images from a patient with preserved and a patient with enhanced spontaneous NET formation are shown in Fig. 1b. Unstimulated NET formation did not differ significantly according to MPN phenotype (Fig. 1c), presence or absence of previous thrombosis (Fig. 1d) and mutational status (Fig. 1e). As hydroxyurea may inhibit certain neutrophil responses, such as P-selectin-induced tissue factor expression^27^, we reasoned that it could prevent the presence of spontaneous NETosis in MPN. However, comparison of hydroxyurea-treated vs. untreated patients revealed no significant differences (Fig. 1f), rendering this possibility unlikely. In addtion, although we previously demonstrated that aspirin inhibits NET formation *in vitro*^28^, no difference in spontaneous NETosis was found according to the use of low-dose aspirin (Supplementary Fig. 1S).

### Basal reactive oxygen species production by MPN neutrophils

**Figure 2:**
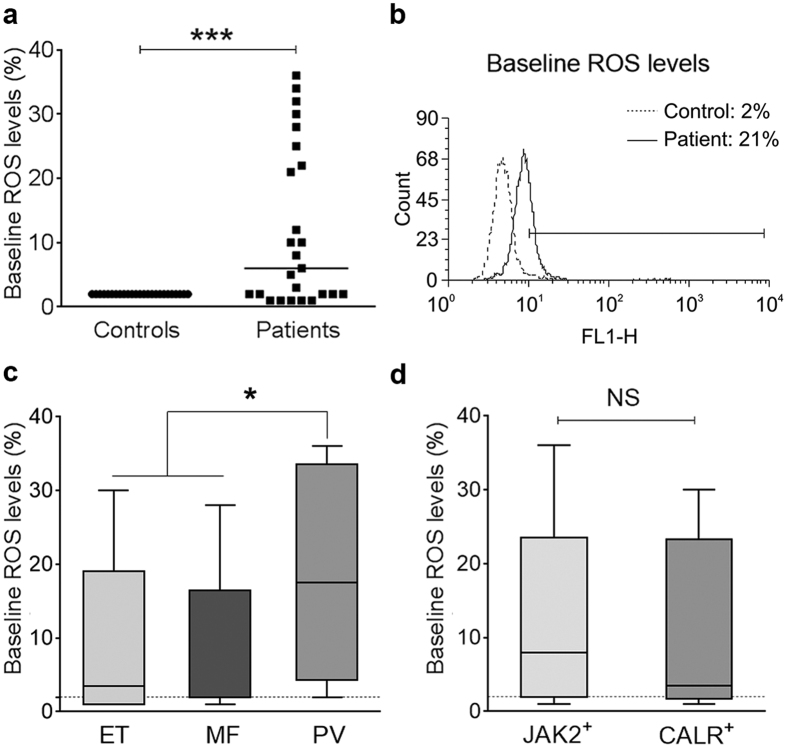
Reactive oxygen species (ROS) production by resting neutrophils. (**a**) Neutrophils were incubated with the fluorogenic probe dihydrorhodamine, ROS production was measured by flow cytometry and expressed as percentage of neutrophils with increased ROS levels compared to a simultaneously studied healthy subject, which was set as 2% to homogeneize measurements performed on different days. ROS levels in patients (n = 25) compared wih controls (n = 25). Horizontal lines represent median values. ***P < 0.001, Wilcoxon matched pairs test. (**b**) Representative histograms showing resting ROS levels in neutrophils from a healthy individual (dashed line) and a patient (continuous line) with increased basal ROS. The percentage of neutrophils with increased ROS production is indicated. (**c**) ROS levels according to MPN phenotype, including ET (n = 8), MF (n = 9) and PV (n = 8). The horizontal dashed line indicates the reference value. Lines in the middle of the box are plotted at the medians, boxes are defined by the 25^th^ and 75^th^ percentiles and whiskers are drawn by the Tukey method. *P < 0.05, Mann-Whitney test. (**d**) ROS production in *JAK2*V617F^+^ (n = 17) vs. *CALR*^+^ (n = 6) patients. The horizontal dashed line indicates the reference value. *P* = NS, Mann-Whitney test.

Increased ROS production induced by JAK2-dependent ERK constitutive phoshorylation and NADPH oxidase activation has been recently described in MPN neutrophils^29^. As ROS generation is a critical intermediate step in NET formation^11^, we assessed basal ROS production and analyzed its correlation with NETosis. The percentage of neutrophils with elevated ROS levels was increased in patients compared to controls (Fig. 2a,b), being higher in PV compared to ET and MF patients (Fig. 2c). ROS production in patients showed a bimodal distribution with a subset showing particularly high (>20%) levels. Interestingly, this group had higher leukocyte counts than those with ROS < 20%, 13.8 (7.5–23.1) vs. 6 (2.5–22.3) x10^9^/L, *P* < 0.05. Of note, elevated ROS was not restricted to *JAK2*V617F^+^ patients, as previously reported^29^, but also involved *CALR*^+^ patients (Fig. 2d). Among patients with increased ROS, 27% had spontaneous NET formation compared to 10% of those with normal basal ROS, *P* = NS. The fact that only one third of patients with high basal ROS showed enhanced spontaneous NETosis suggests that under steady state conditions, higher levels of intracellular ROS or activation of additional signaling pathways are necessary to trigger NET release from MPN cells *ex vivo*.

### NET generation in response to NET inducers

**Figure 3:**
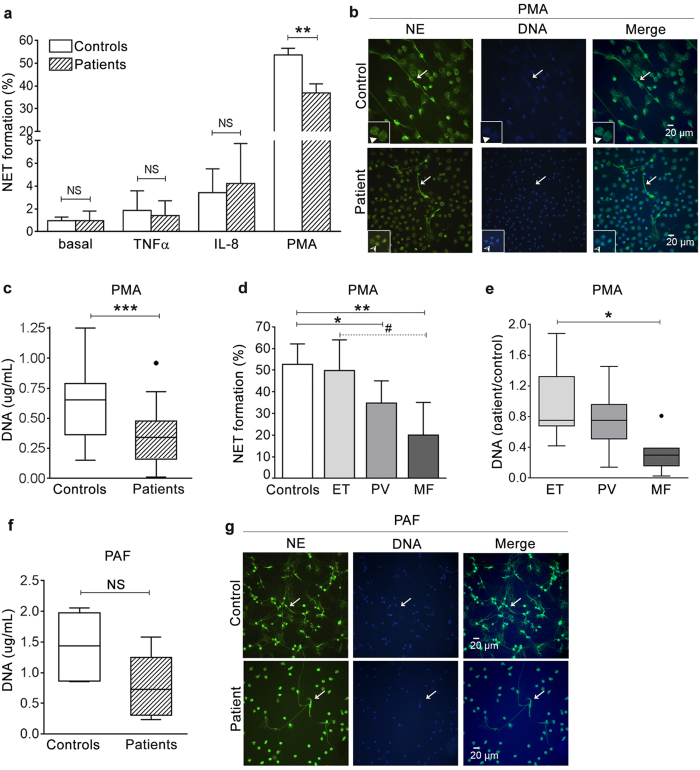
NET formation in response to stimuli. (**a**) Quantification of NETs by microscopy. After stimulation with 20 ng/mL TNFα, 100 ng/mL IL-8 or 50 nM PMA during 180 min., the percentage of neutrophils undergoing NETosis was quantified by fluorescence microscopy in patients (n = 32) and controls (n = 32). Data represent median with interquartile range (for basal, TNFα and IL-8) or mean±SEM (for PMA). *P* = NS, not significant, Wilcoxon matched pairs test; **P < 0.01, paired Student *t*-test. (**b**) Representative images of control and patient neutrophils stimulated with PMA showing decreased NETosis in the latter. Staining was performed with neutrophil elastase (NE)-specific antibody (green) and Hoechst dye (blue). Arrows indicate examples of neutrophils forming NETs. The inset in the upper panel shows neutrophils harbouring enlarged delobulated nuclei with decondensed chromatin (filled arrowheads) and the inset in the lower panel depicts lobulated neutrophils with condensed chromatin (half-filled arrowheads). Original magnification 40x. (**c**) Quantification of PMA-induced NETosis by fluorometry. Patient and control neutrophils were stimulated with 50 nM PMA, NETs were digested with micrococcal nuclease and DNA released to the supernatant was measured by fluorometry. Lines in the middle of the box are plotted at the medians, boxes are defined by the 25^th^ and 75^th^ percentiles. ***P < 0.001, Wilcoxon matched-pairs test. (**d**) Analysis of PMA-induced NET formation by microscopy according to MPN phenotype, including ET (n = 11), PV (n = 10) and MF (n = 11). Data represent median with interquartile range. *P < 0.05; **P < 0.01 vs. controls. ^#^*P* < 0.05 MF vs. ET, Kruskal-Wallis test followed by Dunn’s multiple comparison test. (**e**) Fluorometry of PMA-induced released DNA accross MPN categories, including ET, PV and MF, given as the ratio between DNA released from patient cells and a simultaneously studied control. Lines in the middle of the box are plotted at the medians. *P < 0.05 MF vs. ET, Kruskal-Wallis test followed by Dunn’s multiple comparisons test. (**f**) Fluorometry of 10μM PAF-induced NET formation in patients (n = 6) and controls (n = 6). *P* = 0.06, Wilcoxon matched-pairs test. (**g**) Representative images of control and patient neutrophils stimulated with 10 μM PAF. Examples of neutrophils forming NETs are indicated by the arrows. Original magnification 40x.

Next, we tested the ability of MPN neutrophils to form NETs when stimulated by inflammatory cytokines. Tumor necrosis factor α and IL-8 were used as proinflammatory mediators, as high plasma TNFα and IL-8 levels are prominent features in MPN^8^,^30^. Neutrophils from patients were able to release NETs when stimulated with TNFα and IL-8, and the response to these agents did not differ from controls (Fig. 3a), indicating that MPN cells do not show enhanced NETosis following cytokine stimulation. Considering that under our experimental conditions, TNFα and IL-8 proved to be relatively weak NET stimuli, we assessed the response to PMA, which represents a more potent NET inducer and has been extensively used to characterize NET formation in several disease states^26^,^31^,^32^. Although neutrophils from MPN patients were able to undergo NETosis when stimulated with PMA, this response was significantly reduced compared to controls, as evaluated by assessing the proportion of neutrophils undergoing NETosis by microscopy (Fig. 3a,b) and confirmed by quantification of DNA released to the supernatant by fluorometry (Fig. 3c). Correlation was found between these two methods of NET quantification, r = 0.77, *P* < 0.0001. Impaired PMA-triggered NETosis was most prominent in the subset of patients with MF, as demonstrated by both microscopy (Fig. 3d) and fluorometry (Fig. 3e). Altogether, these findings indicate that although MPN neutrophils are able to form NETs in response to proinflammatory stimuli, a defect in NET generation capacity is revealed when higher levels of NETosis are elicited by more potent inducers, such as PMA. Moreover, MF patients with impaired response to PMA showed a trend towards reduced response to PAF, a lipid mediator with a broad range of effects on the inflammatory response and strong NET inducer^26^,^33^, as revealed by quantification of released DNA (Fig. 3f) and shown in Fig. 3g.

### ROS generation, ERK1/2 phosphorylation and CD11b upregulation triggered by phorbol esther stimulation

**Figure 4:**
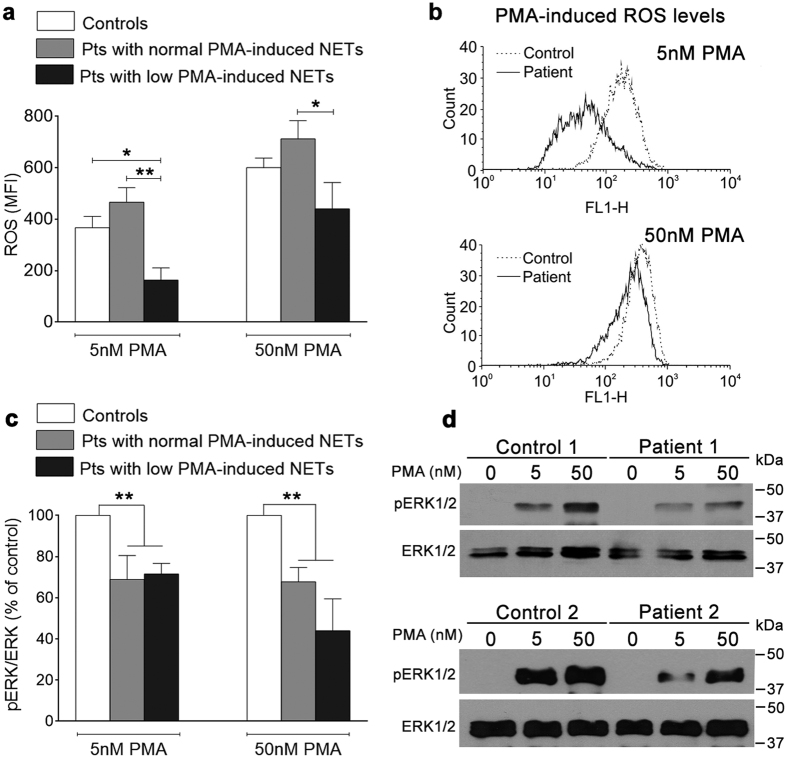
ROS generation and ERK1/2 phosphorylation triggered by PMA. (**a**) Neutrophils were incubated with low (5 nM) and high (50 nM) PMA concentrations during 30 min; then, ROS production was measured by flow cytometry and mean fluorescence intensity (MFI) obtained in unstimulated neutrophils was substracted from MFI obtained after stimulation with PMA. Data represent mean ± SEM of values in controls (n = 21), patients with normal PMA-induced NETosis (n = 14) and patients with reduced PMA-induced NETosis (n = 7, including 6 MF and 1 PV). * *P* < 0.05; **P < 0.01, one-way ANOVA followed by Tukey´s multiple comparison test. Pts. means patients. (**b**) Representative histograms of ROS generation in control neutrophils (dashed line) and in neutrophils from a patient with low PMA-induced NETosis (solid line), showing decreased ROS levels following stimulation with 5 nM and 50 nM PMA. (**c**) After stimulation with 5 nM and 50 nM PMA, neutrophils were lysed and ERK1/2 phoshorylation was measured by Western blot. Protein loading was assessed with total ERK1/2 antibody. Densitometric analysis of band intensity revealed decreased pERK1/2/ ERK1/2 ratio in patients (n = 6) (3 with decreased PMA-induced NETosis and 3 with preserved PMA-induced NETosis) compared to simultaneously studied healthy individuals (n = 6). Data represent mean ± SEM. **P < 0.01, Student *t*-test. (**d**) Representative cropped blots showing decreased pERK1/2 triggered by 5 nM and 50 nM PMA in neutrophils from patients harbouring reduced (patient 1) or preserved (patient 2) PMA-induced NETosis. Full-length blots are presented in Supplementary Fig. 2S.

The signaling cascade underlying PMA-induced NET generation involves PKC-mediated activation of the Raf-MEK-ERK pathway leading to NADPH phosphorylation and ROS production^34^. To assess the mechanisms underlying decreased PMA-triggered NETosis, we measured ROS generation and ERK1/2 phosphorylation in response to this agent. Athough stimulation with 5 nM and 50 nM PMA increased ROS levels vs. baseline in all patients (*P* < 0.0001), those with marked impairment in PMA-induced NETosis (lower than mean minus 2 SD of control values) showed decreased ROS production in response to PMA, whereas ROS levels in patients with normal PMA-induced NETosis were preserved (Fig. 4a,b). These results indicate that low ROS generation capacity may contribute to defective PMA-triggered NET formation found in a subset of MPN patients. Reduced PMA-triggered ERK1/2 phosphorylation was observed in western blots from patients harbouring low PMA-induced NET and ROS generation, suggesting that the signal defect may lie upstream of ROS production. Interestingly, decreased ERK1/2 phosphorylation was not limited to patients with impaired NETosis but was also found in those with preserved NET formation, suggesting that other signaling molecules may compensate for altered ERK1/2 activation in these patients (Fig. 4c,d).

Besides triggering ROS and NET formation, PMA stimulates several other neutrophil functions, including degranulation. As described^5^,^6^, MPN neutrophils (n = 42) showed increased baseline CD11b expression, a marker of degranulation, compared to controls (n = 42); MFI was 24.3 (5.8–108.1) vs. 20.4 (9.5–96.5), *P* < 0.01 (Wilcoxon matched-pairs test). Upregulation of CD11b expression after exposure to 50 nM PMA was preserved in MPN (n = 32) vs. controls (n = 32); fold increase in MFI, 11.2 (2.2–42.9) vs. 12.6 (3.3–37.3), *P* = NS. Adequate CD11b upregulation was also shown for the subgroup of patients with reduced PMA-induced NETosis (n = 9) compared with simultaneousy assayed controls (n = 9); fold increase in MFI, 10.2 (6.1–17.1) vs. 11.8 (4.1–30.6), *P* = NS, indicating that, despite displaying lower levels of PMA-induced NETosis, neutrophil responses to PMA are not globally impaired.

### Circulating nucleosomes in MPN patients

**Figure 5:**
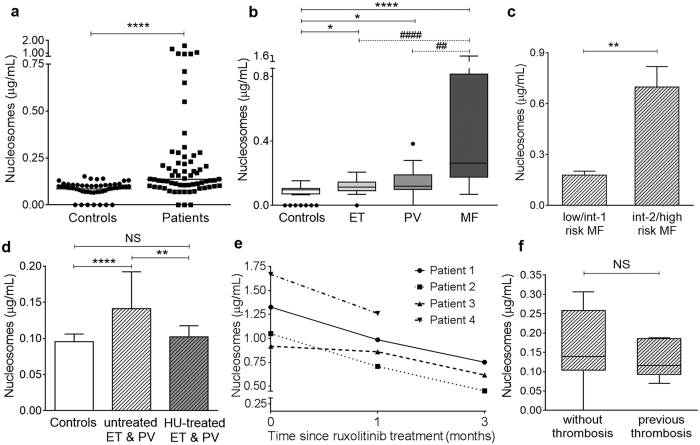
Circulating nucleosomes in MPN patients. (**a**) Nucleosomes were measured in plasma by ELISA. (**a**) Levels in patients (n = 66) compared with controls (n = 52). Horizontal lines represent median values, ****P < 0.0001, Mann-Whitney test. (**b**) Quantification of nucleosomes according to MPN phenotype, including ET (n = 26), PV (n = 15) and MF (n = 25). Lines in the middle of the box are plotted at the medians, boxes are defined by the 25^th^ and 75^th^ percentiles and whiskers are drawn by the Tukey method. *P < 0.05, ****P < 0.0001 vs. controls; ^##^*P* < 0.01 MF vs. PV; ^####^*P* < 0.0001 MF vs. ET, Kruskal-Wallis test followed by Dunn´s multiple comparison test. (**c**) Analysis of MF patients grouped according to DIPSS Plus score in intermediate-2/high risk (n = 14) and intermediate-1/low risk (n = 11). Data are presented as mean ± SEM, **P < 0.01, unpaired Student *t*-test. (**d**) Comparison of untreated (n = 17) vs. hydroxyurea (HU)-treated (n = 24) ET and PV patients. Data represent median with interquartile range. **P < 0.01; ****P < 0.0001; *P* = NS, Kruskal-Wallis test followed by Dunn’s multiple comparison test. (**e**) Decline in nucleosome levels in sequential samples from MF patients during ruxolitinib treatment (n = 4). (**f**) Nucleosome levels according to the presence (n = 16) or absence (n = 50) of previous thrombosis. Lines in the middle of the box are plotted at the medians. *P* = NS, Mann-Whitney test.

Considering that, *in vivo,* NET generation leads to release of DNA complexed with histones to the circulation, we measured plasma levels of nucleosomes, as an indirect measure of NETosis. Circulating nucleosomes were increased in patients compared with controls (Fig. 5a). Analysis of MPN subsets revealed that nucleosomes in all patient groups were significantly higher than in normal subjects; levels were moderately elevated in ET and PV and highest in MF (Fig. 5b). When categorized according to DIPSS Plus score^35^, patients with advanced MF had higher nucleosomes than those with early disease (Fig. 5c) and 3 of 9 patients harbouring levels higher than mean values for MF died within few months after analysis. Whereas no significant difference was found according to mutational status in ET, *CALR*^+^ MF patients showed a trend towards lower nucleosome levels than *JAK2*V617F^+^, 0.22 (0.07–0.71) μg/mL vs. 0.46 (0.08–1.58) μg/mL, *P* = 0.06 (Mann-Whitney test), probably because they tended to cluster in a lower DIPSS Plus category. Comparison of ET and PV patients treated versus those not treated with hydroxyurea revelead that nucleosomes were lower in those under cytoreductive therapy (Fig. 5d), whereas it was not possible to evaluate the effect of treatment in patients with MF owing to patient heterogeneity and the compound effect of risk category. Interestingly, a decline in nucleosome levels was evident in sequential samples from ruxolitinib-treated MF patients (Fig. 5e). No association was found between nucleosomes and the presence or absence of previous thrombosis (Fig. 5f). Levels of VWF antigen, circulating platelet-neutrophil aggregates and D-dimer were measured as markers of endothelial, cellular and coagulation activation. As reported^5^,^6^, increased VWF levels and platelet-neutrophil aggregates were found in this cohort, 7.95 (3.6–28.1) vs. 6.3 (2.4–12.9) μg/mL, *P* = 0.004 and 13.9 (1.5–58) vs. 9.7 (0–23.9)%, *P* = 0.009, respectively (Mann-Whitney test), whereas D-dimer levels were mildly but not significantly increased, 0.27 (0–1.6) vs 0.17 (0–0.51) μg/mL FEU, *P* = NS. No correlation was found between these activation markers and nucleosomes or NETs, *P* = NS for all correlations.

### Plasma MPO, histone-MPO complexes and LDH levels

**Figure 6:**
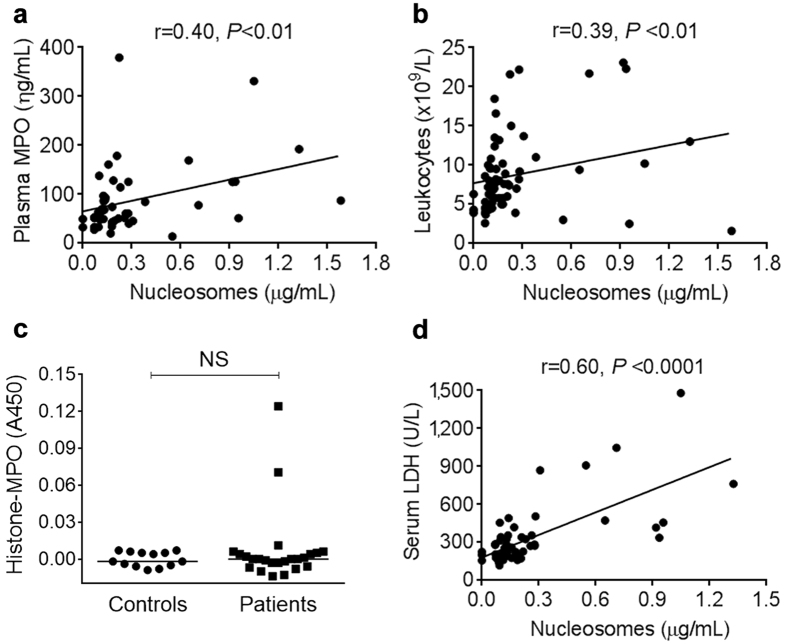
Relationship between nucleosomes, MPO, histone-MPO complexes and LDH. Correlation between circulating nucleosomes with (**a**) plasma MPO and (**b**) leukocyte counts, Spearman correlation. (**c**) Detection of histone-MPO complexes in plasma using a tailor-made ELISA which employs an anti-histone and anti-MPO as the capture and detection antibodies, respectively. Data are given as the difference between absorbance at 450-nm wavelength in samples incubated with the anti-histone Ab and values obtained in duplicate samples omitting the anti-histone Ab. Horizontal lines indicate median values in patients (n = 24, including 14 with MF, 5 with PV an 5 with ET) and controls (n = 13). *P* = NS, Mann-Whitney test. (**d**) Correlation between circulating nucleosomes with serum LDH, Spearman correlation.

Based on the fact that elevated circulating nucleosomes were not associated with enhanced *ex vivo* NET formation and considering that nucleosomes may be originated by mechanisms other than NETosis, we measured plasma MPO, circulating histone-MPO complexes and LDH levels to gain further insight into the source of nucleosomes in MPN. MPO is a major granular protein which represents an integral NET component, although it may also be released as a result of neutrophil degranulation^5^. Plasma MPO was elevated in MPN compared with controls, 66.5 (13.9–379.3) vs 50.9 (17.5–61.9) ng/mL, *P* < 0.05 (Mann-Whitney test), and a certain degree of correlation was found between nucleosomes and both MPO and leukocyte counts (Fig. 6a,b). Recently, complexes of DNA or histones with granular proteins, including MPO or elastase, have been proposed as specific NET biomarkers^13^,^15^,^36^, although there are currently no available standardized assays for measurement of these complexes. Using a tailor-made capture ELISA, we determined histone-MPO complexes in plasma from patients harbouring the highest levels of nucleosomes. No evidence of histone-MPO complexes were found in most plasma samples except for two MF patients, whose levels were above cut-off values (Fig. 6c). The presence of histone-MPO complexes in these two MF patients was confirmed in a separate sample obtained several months apart. Interestingly, one of these patients had a history of prior thrombosis whereas the other had not experienced thrombotic events after a 4-year follow-up. Finally, a stronger positive association was found between nucleosomes and serum LDH, which may be released secondary to cell lysis (Fig. 6d). Altogether, this data suggest that, in most MPN patients, nucleosomes seem not be derived from NETs and that increased cell turn-over may represent a plausible factor contributing to extracellular DNA in this setting.

## Discussion

Although largely beneficial as a host defense mechanism, excessive NETosis may trigger thrombosis and organ damage in diverse conditions characterized by acute or chronic inflammation. Several factors favouring NET release are present in MPN, including neutrophil activation^5^, cross-talk between neutrophils and activated platelets^6^ and a proinflammatory background, enriched in cytokines known to prime NETosis, such as TNFα and IL-8^8^,^30^. Despite confirming the presence of neutrophil activation in this cohort, as evidenced by increased baseline CD11b, enhanced spontaneous *ex vivo* NET formation was not a frequent finding and remained limited to a subset of patients, who did not share distinctive clinical features. Excessive ROS production and oxidative stress have been shown to play a role in MPN pathogenesis^37^,^38^. In accordance with a recent study^29^, we confirmed that MPN neutrophils display increased basal ROS and extended this observation by showing that high ROS levels were not restricted to *JAK2*V617F^+^ patients, as described^29^, but also involved *CALR*^+^ patients. Whereas JAK2-dependent ERK phosphorylation and NADPH oxidase activation underlie ROS generation in *JAK2*V617F^+^ patients^29^, further study will determine whether upregulated JAK2 signaling, which also occurs in *CALR*^+^ patients^39^, is responsible for increased ROS in patients without the *JAK2* mutation. Considering that ROS production is a key intermediate step in NET formation^11^, the fact that, in this study, high basal ROS levels were not uniformly associated with spontaneous NETosis, is intriguing. This may be in line with current data indicating that, although essential for NETosis, ROS production may not be sufficient in its own to trigger it and concomitant stimulation of additional signaling pathways is required^40^.

In this study, no significant differences in neutrophil responses, including NET and ROS generation and CD11b expression at baseline or in response to stimuli was found between untreated patients and those treated with hydroxyurea (as shown in Fig. 1f and Supplementary Table S1), indicating that although hydroxyurea may inhibit certain neutrophil functions^27^, these parameters were not significantly influenced by this cytoreductive treatment.

In an attempt to mimic the inflammatory mileu occurring *in vivo,* we next studied NET production after stimulation with TNFα and IL-8 and found that, in addition to the absence of spontaneous NETosis, patient neutrophils did not show hypersensitivity to these inflammatory mediators. Moreover, when challenged with a stronger NET inducer, such as PMA, MPN cells showed defective NET production. In particular, PMA-induced NETosis was significantly reduced in PV and MF, being most impaired in the latter, whereas the response in ET was preserved. This abnormality might represent an intrinsic cell defect, inherent to the abnormal MPN clone, or be acquired during circulation secondary to chronic *in vivo* activation and subsequent cell exhaustion. We showed that PMA-triggered ROS production was reduced in patients harbouring a profound impairment in NET formation, indicating that this defect lies, at least in part, at the step of ROS production, which may be due in turn to defective signals upstream of ROS, as suggested by the finding of reduced ERK1/2 phosphorylation. A more comprehensive study of other phagocytic functions may help determine whether neutrophil function is altered in MPN, particularly in MF patients, as suggested by previous findings^41^.

A major finding of this study was the presence of elevated circulating nucleosomes. Nucleosomes may be originated by NETosis or by other forms of cell death, such as necrosis or apoptosis^42^. The contribution of NETs to circulating nucleosomes in several clinical conditions is still a matter of debate. Several groups have relied on the identification of MPO or elastase attached to the DNA-histone backbone to establish whether nucleosomes are derived from NETs^13^,^15^,^36^. Histone-MPO complexes were present in a minority of patients in this cohort. Failure to detect these complexes in a large proportion of MPN samples raises the possibility that NETs may not be the main source of nucleosomes in most MPN patients. Therefore, although a few patients showed increased levels of histone-MPO complexes, absence of this feature in most studied patients does not provide evidence for a major role of NETosis in MPN, at least at steady state conditions. Alternatively, it might reflect the inability of the assay to detect lower levels of NETosis, as may occur in chronic conditions, as opposed to higher levels that may be found in acute states, such as septic shock. Availability of standardized methods for the identification of circulating NETs would help to definitively clarify this matter. Considering that clonal myeloid proliferation is a prominent feature of MPN, cell death secondary to increased turn-over may be another potential source of nucleosomes in this setting, as suggested by their association with high LDH. Along this line, elevated nucleosomes have been described in several malignant diseases, such as lymphoma and solid tumours^43^.

Regardless of their origin, nucleosomes may cause harmful effects. Both cell-free DNA and extracellular histones have been shown to activate coagulation, whereas histones may induce endothelial dysfunction and platelet activation^44^,^45^. Although nucleosomes might thus contribute to clot formation, the absence of correlation between nucleosome levels and previous thrombosis or activation markers does not seem to support the use of this parameter to predict thrombosis in MPN. However, this analysis might be limited by the relatively small size of the study population. Longitudinal prospective studies of larger patient cohorts would be required to establish this issue. Considering that, as shown in this work, cytoreductive treatment reduces nucleosome levels, analysis of samples from untreated patients would help to adequately address this matter.

Histones may, in addition, act as DAMPs to promote inflammation and cytokine release through toll-like receptor activation^46^, thus perpetuating the hyper-inflammatory state that characterizes MPN, particularly MF patients. We showed here that levels of nucleosomes varied according to MPN category, being highest in MF, particularly in those with high-risk features, indicating that nucleosomes may be a marker of advanced disease. Prospective studies in a larger MF cohort are necessary to assess the potential prognostic value of nucleosomes in the context of current risk stratification systems. Ruxolitinib has been show to ameliorate inflammation and decrease circulating cytokines in MPN patients^47^. Interestingly, in this study, a reduction in nucleosome levels was shown in sequential samples during ruxolitinib treatment, in parallel with clinical improvement and a decrease in C-reactive protein, a marker of inflammation.

In conclusion, our data show that neutrophils from MPN patients, despite displaying *in vivo* activation and increased basal ROS, are rarely prone to *ex vivo* NET generation and show, in fact, impaired PMA-induced NETosis, particularly in MF. In addition, the finding of elevated circulating nucleosomes was a prominent feature of this study, higher levels being associated with advanced disease, and may represent a potential prognostic marker. Overall, our results do not provide evidence for relevant *in vivo* NETosis under steady state conditions and suggest that increased cell turn-over might be a plausible source of nucleosomes in this setting. However, further research in this field and availability of standardized NET biomarkers may help establish this issue and define the potential contribution of NETs to MPN thrombosis.

## Methods

### Patients

Patients with MPN diagnosed according to the WHO criteria^48^, comprising ET, PV and primary, post-TE and post-PV myelofibrosis (MF), were included after written informed consent. Patients (n = 66) did not differ significantly from controls (n = 52) regarding age, 60 (19–86) vs. 56 (26–85) years old, and sex, 74 vs. 62%, respectively, were women. The study was approved by the Institute of Medical Research “Alfredo Lanari” Ethics Committee. All experiments were performed in accordance with relevant guidelines and regulations.

### Sample preparation

After removal of platelet-rich plasma, neutrophils were purified by Ficoll-Hypaque gradient and dextran sedimentation, as described^14^. Plasma was obtained from EDTA-anticoagulated blood by two sequential centrifugation steps at 2500 × *g* for 15 minutes at 4 °C and stored at −70 °C.

### NET formation by fluorescence microscopy

Neutrophils (2 × 10^5^) were resuspended in RPMI 1640 supplemented with 0.5% fetal bovine serum, seeded in 24-well plates with polylysine-coated coverslips and incubated for 180 minutes, as described^14^, with or without 20 ng/mL TNFα (Peprotech, Rocky Hill, NJ, USA), 100 ng/mL IL-8 (Peprotech), 50 nM PMA (Enzo Life Sciences, Lausen, Switzerland) or 10 μM platelet-activating factor (PAF) (Sigma-Aldrich), fixed and permeabilized. After blocking with 5% goat serum, immunolabeling was performed with anti-neutrophil elastase rabbit pAb (Calbiochem, San Diego, CA, USA), followed by goat anti-rabbit IgG Alexa Fluor 488 (Life Technologies, Eugene, OR, USA) and Hoechst 33258 (Sigma-Aldrich, St. Louis, MO, USA). Images were acquired on an epifluorescent microscope (AXIOSTAR plus Zeiss, HAL 100, Göttingen, Germany). NETs were scored by two independent observers as clouds of DNA emanating from cells that stained positive for both neutrophil elastase and Hoechst and were at least twice as long as the cell diameter or neutrophils showing delobulated enlarged nuclei with conspicuous chromatin decondensation, an early sign of NETosis, as described^26^,^31^,^34^,^49^. Cells in at least 20 non-overlapping fields were counted for each condition and results were expressed as percentage of neutrophils forming NETs.

### Fluorometry of released DNA in NET supernatants

In parallel experiments, neutrophils were placed in 24-well plates without coverslips and allowed to form NETs, as described^14^. After digestion with 500 mU/mL Micrococcal nuclease (Roche, Mannhein, Germany) for 15 minutes at 37 °C, 5 mM EDTA was added and supernatants were collected, centrifuged and stored at −20 °C. DNA was quantified with SYBR Gold (Invitrogen, Carlsbad, CA, USA) in a fluorometer (BioTek Instruments, Winooski, VT, USA). The calibration curve was constructed using thymus DNA of a known concentration.

### Flow cytometry

To measure ROS levels, neutrophils were labeled with 5 μM dihydrorhodamine (Sigma-Aldrich) and incubated with or without PMA at 37 °C for 30 minutes. To assess CD11b expression and platelet-neutrophil aggregates, leukocytes in PBS-diluted blood were labeled with fluorescein isothiocyanate-conjugated anti-CD45 and phycoerythin-conjugated anti-CD11b or -CD41 antibodies (Biolegend San Diego, CA, USA), or corresponding isotype controls, during 15 min. with or without 50 nM PMA. Cells were analyzed in a flow cytometer. The neutrophil population was selected based on CD45 expression and side scatter (SSC) and platelet-neutrophil aggregates were identified as the percentage of events staining positive for CD41. Baseline CD11b was calculated as the ratio between mean fluorescence intensity (MFI) of antibody staining and the isotypic control. Fold-increase in CD11b was defined as the ratio between MFI of the stimulated sample minus baseline CD11b and baseline CD11b.

### ERK1/2 phosphorylation by Western blot

Neutrophils (6 × 10^6^) were incubated with or without PMA for 45 minutes at 37 °C, lysates were prepared with RIPA buffer and 40 μg proteins were resolved by SDS-PAGE. Immunoblotting was performed with mouse anti- p-ERK1/2 (Tyr 204) (Santa Cruz Biotechnology, Dallas, TX, USA) and protein loading was assessed with rabbit anti-ERK1/2 followed by the corresponding HRP-conjugated secondary antibodies and detection by enhanced chemiluminiscence.

### Quantification of nucleosomes, circulating myeloperoxidase (MPO), histone-MPO complexes, VWF and D-dimer levels

Nucleosomes were measured in plasma using Cell Death Detection ELISA (Roche). A standard curve was prepared by making serial dilutions of a DNA-histone complex of a known concentration, supplied by the manufacturers. MPO levels were determined by LEGEND MAX™ ELISA (Biolegend) and histone-MPO complexes by ELISA, as described^50^, with modifications. Briefly, biotinylated anti-histone antibody (1:25 dilution) was added to streptavidin-coated plates (Cell Death Detection) and incubated during 2 hours. After blocking with 2.5% bovine serum albumin, plasma samples (1:4 dilution) were incubated during 2 hours followed by 2-hour incubation with HRP-conjugated anti-MPO antibody (Quantikine ELISA Human MPO, R&D, Minneapolis, MN, USA) and addition of tetramethylbenzidine, all at room temperature. Identical procedure was carried out for each sample omitting the anti-histone antibody. Absorbance at 450-nm wavelength of samples without anti-histone antibody (non-specific signal) was substracted from that of duplicate samples with anti-histone antibody (histone-MPO complexes). Supernatant of PMA-stimulated and unstimulated neutrophils was used as positive and negative control for NETosis, respectively. Antigen levels of VWF were measured by ELISA, as described^51^ and D-dimer by immunoturbidimetric assay (Innovance D-dimer, Siemens, Erlanger, Germany).

### Statistical analysis

Data were tested for Gaussian distribution. For comparison between two groups, unpaired Student´s *t*-test was used for normally distributed data and Mann-Whitney test was applied for non-normally distributed data. For comparison among multiple groups, ANOVA with Tukey´s post-test or Kruskal-Wallis with Dunn´s post-test were used in case data followed a normal or non-normal distribution, respectively. Student´s paired *t*-test or Wilcoxon matched pairs test were applied for analysis of control and patient samples assayed simultaneously, according to whether or not variables followed a Gaussian distribution, respectively. Categorical values were examined by Fisher´s exact test and correlations with the Spearman correlarion test. All statistical analyses were two-sided and *P* values < 0.05 were considered significant. The GraphPad Prism 6.01 (La Jolla, CA, USA) software was applied.

## Additional Information

**How to cite this article**: Marin Oyarzún, C. P. *et al*. Neutrophil extracellular trap formation and circulating nucleosomes in patients with chronic myeloproliferative neoplasms. *Sci. Rep.* **6**, 38738; doi: 10.1038/srep38738 (2016).

**Publisher's note:** Springer Nature remains neutral with regard to jurisdictional claims in published maps and institutional affiliations.

## Supplementary Material

Supplementary Information

## Figures and Tables

**Table 1:** Cinical feaures of patients with myeloproliferative neoplasms at the time of the study.

	ET	PV	MF
Number of patients	26	15	25
Age (years)	49 (22–84)	64 (45–80)	59 (19–86)
Female, n (%)	21 (80.7)	10 (66.6)	18 (69.2)
Prior thrombosis, n (%)*	7 (27)	5 (33)	4 (16)
*JAK2* V617F^+^, n (%)	17 (65)	15 (100)	12 (48)
*CALR*^+^, n (%)†	6 (23)	—	11 (44)
Triple-negative, n (%)†	3 (12)	—	2 (8)
Cytoreductive treatment, n (%)‡	13 (50)	11 (73.3)	11 (44)
Patients receiving aspirin, n (%)	15 (58)	10 (66.6)	11 (44)
Time since diagnosis (months)	118 (2–328)	76 (1–277)	126 (3–291)

ET means essential thrombocythemia; PV, polycythemia vera; MF, myelofibrosis, and includes 15 patients with primary MF, 5 with post-ET and 5 with post-PV MF. Values are reported as median and range.

^*^Thromboembolic events included deep vein thrombosis (n = 3), pulmonary embolism (n = 2), splachnic vein thrombosis (n = 1), stroke (n = 3), acute myocardial infarction (n = 3), transient ischemic attack (n = 3), peripheral arterial disease (n = 2) and ischemic colitis (n = 1). Elapsed time from the thrombotic episode to inclusion in this study was 62 (1–264) months.

^†^Screening for *JAK2*V617F, *CALR* and *MPL* mutations was perfomed as described^52^,^53^,^54^. *CALR* and exon 10 *MPL* mutations were studied in *JAK2*V617F-negative patients. No *MPL* mutations were detected.

^‡^Cytoreductive treatment consisted of hydroxyurea in ET and PV and included hydroxyurea (n = 4), pegylated alpha interferon (n = 4) and ruxolitinib (n = 3) in MF patients. At the time of the study, 3 patients received acenocoumarol and 1 was treated with low-molecular weight heparin.
