# Supplementary material for: Neutrophil extracellular trap formation and circulating nucleosomes in patients with chronic myeloproliferative neoplasms
Source: Sci Rep. 2016 Dec 13;6:38738. doi: 10.1038/srep38738 (PMC5153854; doi:10.1038/srep38738)

Neutrophil extracellular trap formation and circulating nucleosomes in patients with chronic myeloproliferative neoplasms

Cecilia P. Marin Oyarzún,[1] Agostina Carestia,[2] Paola R. Lev,[1] Ana C. Glembotsky,[1] Miguel A. Castro Ríos,[3] Beatriz Moiraghi,[4] Felisa C. Molinas,[1] Rosana F. Marta,[1] Mirta Schattner[2*] and Paula G. Heller[1*]

Supplementary Table S1. Comparison between untreated and hydroxyurea (HU)-treated patients.

	Untreated	HU-treated	*P*
Baseline ROS levels (%)	12 (1-34)	3.5 (1-36)	NS
Baseline CD11b expression (MFI)	26.1 (9.6-66.6)	23.4 (11.9-98.2)	NS
PMA-induced NET formation (%)	43.4 ± 25.6	32.6 ± 17.4	NS
5 nM PMA-induced ROS production (MFI)	308 (77-596)	323.5 (10-870)	NS
50 nM PMA-induced ROS production (MFI)	525 (385-1096)	559.5 (51-1128)	NS
PMA-induced CD11b (fold-increase in MFI)	9.8 (4.7-42.9)	12.5 (2.2-27.1)	NS

Data are presented as median and range or mean ± standard deviation, as appropriate, and analyzed by Mann-Whitney test or unpaired student t test, respectively.

ROS means reactive oxygen species; MFI, mean fluorescence intensity; NET, neutrophil extracellular trap.

Supplementary Figure S1. Unstimulated NET formation, as evaluated by fluorescence microscopy, in patients with myeloproliferative neoplasms receiving (n=19) or not (n=13) aspirin. Lines in the middle of the box are plotted at the medians, boxes are defined by the 25th and 75th percentiles and whiskers are drawn by the Tukey method. NS= not significant, Mann-Whitney test.

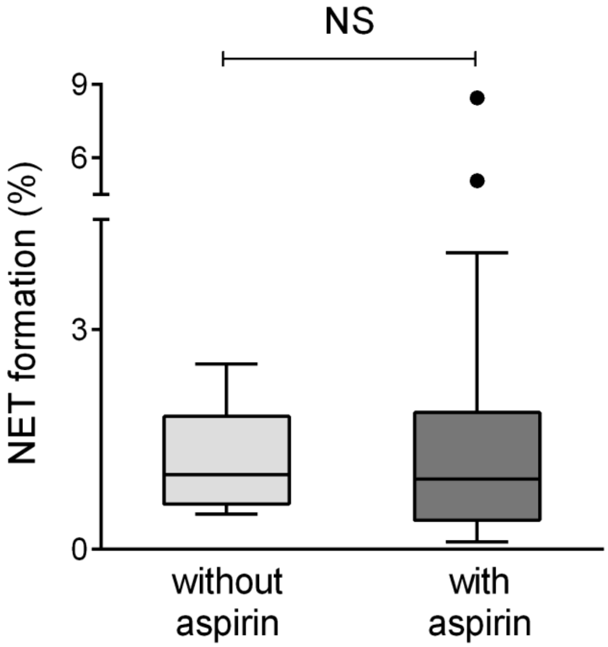

Supplementary Figure S2. Representative full-length blots showing decreased pERK1/2 triggered by 5nM and 50nM PMA in neutrophils from patients harbouring reduced (patient 1) or preserved (patient 2) PMA-induced NETosis.

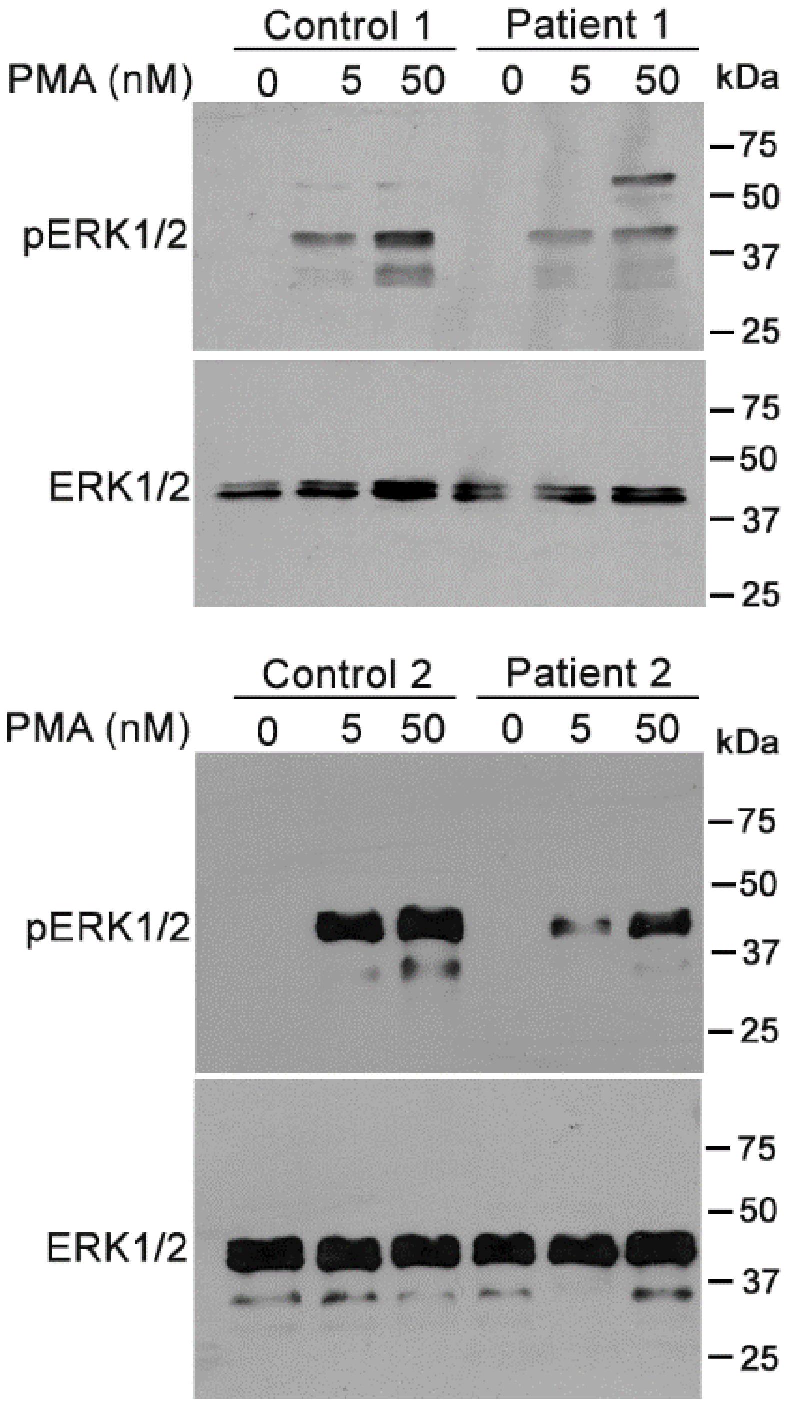

Supplement: Supplementary Information [file srep38738-s1.pdf]
